# Health complaints among adolescents in Norway: A twenty-year perspective on trends

**DOI:** 10.1371/journal.pone.0210509

**Published:** 2019-01-09

**Authors:** Thomas Potrebny, Nora Wiium, Anne Haugstvedt, Ragnhild Sollesnes, Torbjørn Torsheim, Bente Wold, Frode Thuen

**Affiliations:** 1 Centre for Evidence-Based Practice, Faculty of Health and Social Sciences, Western Norway University of Applied Sciences, Bergen, Norway; 2 Department of Health Promotion and Development, Faculty of Psychology, University of Bergen, Bergen, Norway; 3 Department of Psychosocial Science, Faculty of Psychology, University of Bergen, Bergen, Norway; 4 Department of Health and Caring Sciences, Faculty of Health and Social Sciences, Western Norway University of Applied Sciences, Bergen, Norway; University of Illinois at Chicago College of Medicine, UNITED STATES

## Abstract

**Purpose:**

Examine time trends in health complaints among adolescents in Norway between 1994 and 2014 and among population subgroups, e.g., age and gender, as well as their interactions.

**Methods:**

Norwegian data on 11-16-year-olds were drawn from the Health Behaviour in School-aged Children survey (HBSC) and analyzed for 1994 (*n* = 4,952), 1998 (*n* = 5,026), 2002 (*n* = 5,023), 2006 (*n* = 4,711), 2010 (*n* = 4,342) and 2014 (*n* = 3,422). Design adjusted linear regression that accounts for clustering effects was used to examine mean scores of two subscales of the HBSC-symptom checklist: psychological and somatic health complaints.

**Results:**

Psychological and somatic health complaints among adolescents in Norway followed somewhat different trajectories, but the mean scores of both types of health complaints appeared to increase during the 20-year period. For psychological health complaints, there was a three-way interaction between age, gender and time, indicating that increasing trends in health complaints depended on both age and gender, in which older adolescent girls had a greater increase over time relative to younger adolescents and boys.

**Conclusions:**

Findings from this study, together with earlier findings, suggest that there has been an increasing trend in health complaints among adolescents in Norway from 1994 to 2014, especially among older adolescent girls. Future research should examine if trends in health complaints also depend on gender and age in other contexts. This will help the planning and implementation of tailored and effective interventions.

## Introduction

At present, there are increasing concerns about reports of deteriorating mental health in non-clinical youth populations from high-income countries between the 1950s and 2016 [1–4]. One highlighted indicator is recurring health complaints among young people, which is considered to be an important indicator of subjective well-being, as it reflects individual burden and personal experience related to negative life events in the social context of family, school and peers [5]. Health complaints consist of the subjective experience of psychological (mental health complaints) and somatic (recurring pains and aches) symptoms, without any presumption of underlying mental illness [5, 6], even though recurring health complaints are recognized as important indicators of mental health in adolescence [7]. These subjective health complaints are also referred to as “psychosomatic complaints” in the literature.

Northern Europe appears to have had the greatest deteriorating trend in health complaints compared to other regions [2, 8]. In a comparative study involving countries in Europe and North America, adolescents from the Nordic countries seem to particularly stand out with indications of increasing health complaints over recent decades, even though the prevalence of two or more weekly health complaints among 15-year-olds is generally low in most of the Nordic countries compared to other regions [8]. The Nordic increase in health complaints occurred despite these countries ranking very high on the Human Development Index (HDI) [9] and holding a leading position in promoting health through public policy [10] which, in some respects, represents a public health puzzle. Norway is highlighted as the country with the largest increase between 1994 and 2010, at a rate of almost 11% (from 21.8% to 32.5%). In contrast, a decline in health complaints has been observed in North America, for example the United States, where a sharp 9% decrease (from 45.7% to 39.6%) between 1998 and 2010 was observed [8]. This might suggest that even though the rates of recurring health complaints are generally lower in the Nordic countries compared to other regions, the Nordic countries may be converging toward regions that are known to have higher rates of health complaints among adolescents, the cause of which is currently unknown.

To assess the time trends in subjective health complaints in Norway, we performed a literature review of empirical evidence, Norwegian grey literature and ongoing Norwegian projects tracking trends in health complaints among school-aged children. The available empirical findings suggest an increase in health complaints (although small) between 1992 and 2010 among 11-17-year-olds in Norway [8, 11–13]. National population registries on adolescent health, such as “Ungdata” [14], report relatively stable levels of health complaints from 2011 to 2016 among adolescents aged 13–19. However, there are indications of divergent trends, in which boys show rather stable levels of complaints, while girls show a small increase. The Norwegian Institute of Public Health [15] survey on living conditions (“Norhealth”) indicates an increase in psychological complaints between 1998 and 2012 among 16-24-year-olds. Although a rise in health complaints is observed in both genders, the strongest increase is found among young girls, with a high rate of complaints that increased from 13% in 1998 to 23% in 2012, compared to 7% in 1998 to 12% in 2012 for young boys. Thus, while there is clear evidence that girls generally have more health complaints than boys in absolute terms, the evidence of different trends over time for gender is inconclusive [2], although there are some indications of a greater increase among girls over time [16–20]. In other Northern European countries, it has also been found that gender differences in health complaints depend on the age of the adolescents [21]. Nonetheless, there appears to have been a clear rise in health complaints among young people, aged 11–24, living in Norway between 1992 and 2016.

A Norwegian prospective study has associated high levels of health complaints among adolescents with high school dropout [22]. Results from the same cohort of adolescents also indicate that adolescents who do not finish secondary education have an increased risk of sickness and disability in the transition to young adulthood [23]. Such findings illustrate why both high school dropout and health complaints among adolescents are considered to be pressing public health issues in their own right [7, 22].

Although health complaints among generational cohorts of adolescents in Northern Europe, including Norway, seem to be increasing, the evidence is not conclusive and there are still unanswered questions to be addressed. Thus, there is a highlighted need for high quality research on trends in adolescent health [1].

The present study aims to analyze Norwegian trends in adolescent health complaints over twenty years, between 1994 and 2014. The study will also examine trends among gender, age and time as well as their interactions, while accounting for the complex sampling procedure in our study.

## Method

### Sample

The data analyzed were drawn from the Health Behaviour in School-Aged Children (HBSC), a World Health Organization collaborative cross-national project tracking health, health behavior, school and social factors among a nationally representative sample of students in primary school, (Norwegian grade 6) consisting of 11–12 year olds and lower secondary school (Norwegian grades 8 and 10) consisting of 13–16 year olds. The HBSC was first carried out in 1983 and included samples from five countries. Since then, the survey has been conducted every four years and now 48 countries in Europe and North America take part. All countries partaking in the project follow a standardized protocol.

This study focused on the full sample of Norwegian adolescents in which health complaint items were comparable. This included six survey waves from the following years: 1993/1994, 1997/1998, 2001/2002, 2005/2006, 2009/2010 and 2013/2014. The number of respondents in each wave was 4,952 (49% girls), 5,026 (49% girls), 5,023 (49% girls), 4,711 (48% girls), 4,342 (50% girls) and 3,422 (51% girls), respectively, resulting in a total sample of 27,476 adolescents. The response rates at the student level for each survey cycle were 82%, 93%, 88%, 85%, 81% and 76%, respectively. The participating students were nested within school classes, thus necessitating adjustments for cluster effects. There were 141, 288, 254, 277, 208 and 284 primary sampling units (school classes), respectively, for the six waves of survey. Design-weights were created using the primary sampling units, stratified by survey year. This design-weight provide valid and robust standard errors in further regression analyses [24–26].

### Procedure

The selection process followed a randomized clustered sampling strategy based on national registries of schools and school classes with the aim of having nationally representative samples. Parents, school administrators and teachers were informed of the study in advance. Passive consent was obtained from parents and school staff, who were offered the opportunity to decline participation. All students were informed that their participation in the study was voluntary and assured anonymity of their responses. For the schools that wanted to participate, teachers supervised the students as they completed the questionnaires in their usual classrooms. Students who did not participate in the survey were either absent from school or attending schools for children with special needs [5].

Norwegian data collection was approved by NSD-Norwegian Centre for Research Data. Additional information about the data collection process can be found elsewhere [5].

### Measures

The HBSC Symptom Checklist (HBSC-SCL) was used to measure the adolescents’ subjective health complaints. The HBSC-SCL is a non-clinical measure consisting of eight health complaint items: headache, abdominal pain, backache, feeling low, irritability or in a bad mood, feeling nervous, sleeping difficulties and dizziness. Adolescents were asked how often they experienced these symptoms over the last six months. The five response categories were: “about every day”, “more than once a week”, “about every week”, “about every month” and “rarely or never”. Previous research found that the HBSC-SCL has adequate test-retest reliability and validity properties [27]. Other research has shown support for a two-factor solution for the HBSC-SCL: a dimension of psychological health complaints (feeling low, irritability or in a bad mood, feeling nervous and sleeping difficulties) and a dimension of somatic health complaints (headache, abdominal pain, backache and dizziness) [21, 28]. Before the analysis, the mean score for each of the two dimensions was calculated. The mean scores were obtained by adding the items scores from 0 (“rarely or never”) to 4 (“about every day”) and dividing by the number of items in the dimension. Of those with valid scores, roughly 97.5% answered all the questions.

### Data analysis

Statistical analysis was performed using the “lavaan” [29] and “survey” packages [26] in R [30]. In the first step, the lavaan package was used to perform a confirmatory factor analysis (CFA) in order to test the underlying factor structure of the HBSC-SCL. A unidimensional factor model was compared to a model comprised of two correlated factors (psychological and somatic) in order to determine which model(s) fit the data best. When considering model fit, it is good practice to assess more than one goodness-of-fit indices [31]. The following goodness-of-fit indices are reported: the model chi-square, Comparative Fit Index (CFI), the root mean square error of approximation (RMSEA) and the Standardized Root Mean Square Residual (SRMR). Values of chi-square p > 0.05 (sensitive to a large sample size), CFI > 0.95, RMSEA < 0.05 and SRMR < 0.08 indicate a good model fit [31]. Robust maximum likelihood estimators were used.

In the second step, the “survey” package, developed for analyzing complex survey samples, was used to model age, gender and time differences by conducting a linear regression analysis that accounted for the design effect of the HBSC’s sampling procedure. Schnohr et al. have recommended accounting for complex sampling in analysis of HBSC data [32]. Age and time were centered at their first values (age 11 and year 1994), making the regression coefficients meaningful and easier to interpret. The time-coefficient represent the annual change of health complaints among adolescents in the study period between 1994–2014. Statistically significant changes over time will be referred to as the “time trend” or “trend”.

Trends and trends by subgroup interactions were tested using Wald F-test for complex survey designs. The Wald-F statistic was used for testing the null hypothesis that the regression coefficient of an independent variable in the model is not significantly different from zero. If the test fails to reject the null hypothesis, removing the variable from the model will not substantially reduce the fit of that model. In analysis of complex samples, the Wald test degrees of freedom is corrected to encompass the survey design [24, 25]. Only interactions that significantly improved the model fit were included in the final analysis.

## Results

### Preliminary analysis

To confirm previous recommendations of a two-factor structure of health complaints, a design-based CFA accounting for complex samples was performed to assess the model fit in the Norwegian sample. The two-factor model showed a good fit based on several goodness-of-fit indices (X^2^ = 602.78, df = 19, p<0.001; CFI = 0.976; RMSEA = 0.041; SRMR = 0.021) and indicated a superior fit to the data in comparison with a unidimensional model (X^2^ = 2059.44, df = 20, p<0.001; CFI = 0.929; RMSEA = 0.076; SRMR = 0.036), in line with previous findings [21, 28]. The findings from the CFA supported treating psychological and somatic complaints as two separate scores in the subsequent analysis.

### Descriptive statistics

Summarizing the unadjusted raw trends in psychological and somatic health complaints over a period of twenty years (1994–2014), both types of complaints appear to have increased from 1994 to 2010, followed by a slight decrease between 2010 and 2014. Table 1 shows the development of psychological health complaints among adolescents by gender and time. Descriptively, boys had overall lower levels of psychological health complaints compared to girls. Furthermore, boys had an increase in psychological health complaints between 1994 and 2010, followed by a decrease in 2014. Girls had higher levels of psychological health complaints than boys at all time points; their scores increased from 1994 to 2010, then decreased in 2014.

**Table 1:** Unadjusted psychological and somatic health complaints by gender and time.

		Mean (95% CI)					
	Year	1994	1998	2002	2006	2010	2014
Psychological	Boys	0.88 (0.85, 0.92)	0.86 (0.83, 0.89)	0.96 (0.92, 0.99)	0.99 (0.95, 1.03)	0.99 (0.95, 1.03)	0.87 (0.83, 0.92)
	Girls	1.07 (1.03, 1.11)	1.06 (1.03, 1.10)	1.24 (1.20, 1.28)	1.21 (1.16, 1.26)	1.34 (1.29, 1.38)	1.23 (1.16, 1.29)
Somatic	Boys	0.55 (0.52, 0.57)	0.55 (0.52, 0.58)	0.57 (0.53, 0.60)	0.58 (0.55, 0.62)	0.64 (0.61, 0.68)	0.58 (0.54, 0.62)
	Girls	0.76 (0.72, 0.79)	0.82 (0.78, 0.86)	0.88 (0.84, 0.92)	0.82 (0.78, 0.86)	0.9 (0.87, 0.93)	0.84 (0.79, 0.88)

With regard to somatic health complaints, Table 1 shows the development by gender and time. Overall, somatic health complaints scores were lower than psychological health complaint scores. Boys had lower levels of somatic health complaints compared to girls. Like psychological health complaints, somatic health complaints increased for both boys and girls between 1994 and 2010, thereafter decreasing from 2010 to 2014.

### Regression analysis

Table 2 shows the results of the design adjusted linear regressions models. When examining the main effect model, the results indicated an increasing trend in somatic health complaints among adolescents between 1994 and 2014. In addition, girls appeared to have more somatic health complaints compared to boys and older adolescents had more somatic health complaints than younger adolescents. The interaction model, however, indicated a significant two-way interaction for gender by age (*F* (1, 1436) = 77.10, p < 0.001), suggesting that girls report more somatic health complaints as they get older compared to boys. This gender by age effect did not change significantly over time. Furthermore, there was a significant two-way interaction for age by time (*F* (1, 1436) = 4.41, p < 0.05), indicating that older adolescents experienced a greater increase in somatic health complaints than younger adolescents between 1994 and 2014.

**Table 2:** Linear regression analysis assessing trends in health complaints among adolescents in Norway.

	Psychological health complaints				Somatic health complaints			
	Model 1a		Model 1b		Model 2a		Model 2b	
	B (95% CI)	P-value	B (95% CI)	P-value	B (95% CI)	P-value	B (95% CI)	P-value
Girl	0.260 (0.238, 0.282)	**0.001**	0.134 (0.067, 0.202)	**0.001**	0.261 (0.242, 0.280)	**0.001**	0.126 (0.082, 0.169)	**0.001**
Age	0.024 (0.017, 0.032)	**0.001**	-0.017 (-0.031, -0.002)	**0.02**	0.044 (0.038, 0.050)	**0.001**	0.010 (-0.001, 0.021)	0.06
Time	0.008 (0.006, 0.010)	**0.001**	-0.001 (-0.005, 0.004)	0.78	0.004 (0.003, 0.006)	**0.001**	0.001 (-0.002, 0.004)	0.38
Girl:Age			0.018 (-0.004, 0.040)	0.10			0.050 (0.039, 0.061)	**0.001**
Girl:Time			-0.001 (-0.006, 0.005)	0.87			0.001 (-0.002, 0.004)	0.46
Age:Time			0.002 (0.001, 0.003)	**0.02**			0.001 (0.001, 0.002)	**0.04**
Girl:Age:Time			0.004 (0.002, 0.006)	**0.001**				
Constant	0.793 (0.762, 0.825)	**0.001**	0.937 (0.889, 0.985)	**0.001**	0.424 (0.398, 0.449)	**0.001**	0.514 (0.479, 0.550)	**0.001**

Models 1a and 2a are main effect models; Models 1b and 2b are interaction models.

**Fig 1:**
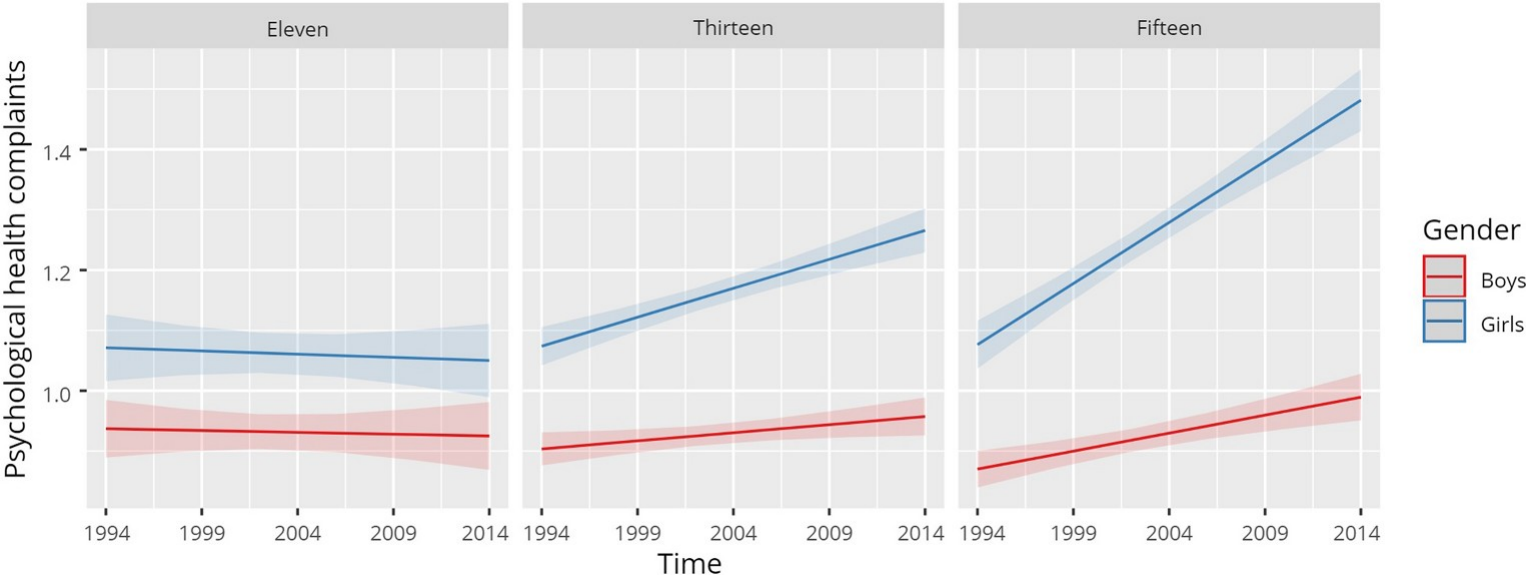
Marginal effects for psychological health complaints by gender and time, grouped by age (mean score, 0–4).

Based on the results in Table 2, psychological health complaints appeared to have a somewhat different trend than somatic health complaints. First, like somatic health complaints, results from the main effect model indicated an increasing trend and higher levels of complaints among girls and older adolescents. Second, unlike somatic health complaints, the interaction model indicated a significant three-way interaction between gender, age and time (*F* (1, 1435) = 13.77, p < 0.001), suggesting that older adolescent girls had the greatest increase in psychological health complaints over time. This finding on older adolescent girls is relative to boys, who had a rather stable trend in psychological health complaints between 1994 and 2014, with only small differences between younger and older adolescent boys (Fig 1).

## Discussion

The trends in psychological and somatic dimensions of health complaints differed to some extent in our analyses. On the one hand, somatic health complaints appeared to be rising for boys and even more so for girls; older girls had the greatest increase, while the gender by age effect did not change over time. On the other hand, psychological health complaints appeared to be more prevalent and increasing at a greater rate over time than somatic health complaints. In addition, a three-way interaction between gender, age, and time suggested that psychological complaints increased mainly among older adolescent girls compared to younger girls and boys over the 20-year period, indicating a divergent trend. Other Norwegian studies report similar findings and indicate an increase in psychological health complaints among adolescent girls between 1992 and 2016 [14, 15], thus supporting the present findings.

Earlier studies from Europe and North America have observed an age or a gender effect on health complaints, but few have investigated a possible three-way interaction that includes change over time. A systematic review of gender and age-specific trends in internalizing problems reported an increase mainly among older adolescent girls over time [1]. Furthermore, previous studies on HBSC data that investigated psychological health complaints in Sweden and Switzerland found that in Sweden, there was a clear increase over time for older girls, while the increase was only minor in Switzerland [21, 33]. For somatic health complaints, Dey et al. [21] also found an increasing trend, but the study did not include tests of a three-way interaction between gender, age and time. Moreover, somatic symptoms during adolescence have been shown to predict adult mental illness even when controlling for adolescent depression, suggesting that adolescents with reoccurring somatic symptoms may need early treatment for these specific symptoms [34]. Somatic and psychological symptoms are also associated with adolescent loneliness [35]. Therefore, high levels of somatic complaints at a young age can be an important early indicator of underlying mental health problems among adolescents.

It is a challenge to elucidate the changing trends in health complaints among adolescents, as (1) our data does not allow any causal inference and (2) evidence indicates that several factors associated with health complaints have changed over the last twenty years, while other determinants have remained stable (such as smoking, being bullied and school-related stress) [12]. It is also known that the timing of puberty contributes to acute health complaints in this age group. It has been speculated that an observed decrease in the age at onset of puberty over the last century may be putting early maturing adolescents at risk of adjustment difficulties, including psychopathology [36, 37]. In particular, early puberty in girls may be indicative of future psychological problems, although it is still uncertain whether this effect is transient or sustained [36]. It has been hypothesized that a rise in psychological health complaints may, to some extent, be explained by an increased willingness to report symptoms. However, an increased willingness to report symptoms is not considered to be a key explanatory factor of growing mental health problems [1]. In fact, some qualitative research suggests that adolescents may just as well avoid disclosing symptoms of mental health problems due to stigma from peers, parents and teachers and to avoid being perceived as “weird” [38, 39]. This might be the case for both girls and boys.

In regard to boys, reporting overall lower health complaints might reflect some differences in reacting to symptoms of mental distress in subtle ways; boys may tend to externalize mental distress, e.g., delinquency or self-medication to a greater extent, allowing distress to build before “blowing off steam” [40]. Ridge et al. [40] highlight that little is known about how men experience mental distress. A study of adult twins from the US [41] does indicate that latent differences in the prevalence of common mental disorders such as internalizing and externalizing syndromes may well be accounted for by gender, indicating higher levels of internalizing and lower levels of externalizing syndromes among women, while the opposite appears to be true for males. The authors note that it is currently uncertain if gender differences in internalizing and externalizing syndromes pertains to the adolescent population as well. Another study does however suggest that the trend in externalizing syndromes appears to be stable over time for both boys and girls, whilst internalizing syndromes appears to be increasing among adolescent girls [1].

Some limitations of this study should be noted. First, pupils who were absent on the day of data collection were not represented. Previous research suggests this may introduce a bias, because adolescents with more health complaints are more likely to be absent from school than adolescents with less complaints [42]. Second, using repeated cross-sectional data allows the analysis of time trends, but does not permit drawing strict causal inferences. Third, from a gender perspective, it should be noted that externalizing behaviors were not included in the study, as the HBSC-SCL mainly consists of internalizing mental health symptoms. As a result, no comments about period effects in externalizing symptoms can be made. Fourth, the trend analysis did not control for socio-economic background, SES has been shown to be a predictor of health in previous studies, where families in lower SES households tend to have poorer health [43]. Previous research has however indicated that SES among adolescents in Norway has remain constant between 1994–2010 [44], although other researchers have questioned the validity and reliability of this specific SES indicator over time [21]. It is therefore unlikely that there have been any substantial changes in health inequalities among adolescents that could confound the trends observed in our findings. Fifth, from a methodological perspective, it appears that the upward trends in health complaints might not be completely linear, which is evidenced by comparing observed values to predicted values in our sample. Observed values indicated a small decrease between 2010 and 2014, making the predicted increase between these two time points more uncertain. However, another Norwegian study does indicate that there has been a yearly increase between 2011 and 2016 among girls, while psychological health complaints among boys remained stable [14], parallel to our main findings and might suggest that the decrease seen in the observed values from 2010 to 2014 could reflect random fluctuations. Any indication of a continued decrease should be followed closely when analyzing future Norwegian data.

## Conclusion

Based on the present findings, there has been an increase in psychological and somatic health complaints among adolescents in Norway, especially among older adolescent girls. This is supported by evidence from Norwegian adolescent population health data that indicate an increase in the prevalence of health complaints among cohorts of young people in Norway between 1994 and 2014, which rightfully confirms that increasing health complaints is a public health concern. To further investigate potential determinants of health complaints, longitudinal studies and continued tracking of health trends are needed. In addition, intervention strategies that will assist adolescents in managing psychological and somatic health complaints are vital.
